# Prevalence and morbidity of urogenital schistosomiasis among pre-school age children in Cubal, Angola

**DOI:** 10.1371/journal.pntd.0011751

**Published:** 2023-11-08

**Authors:** Raquel Sánchez-Marqués, Cristina Bocanegra, Fernando Salvador, Arlette Nindia, Zeferino Pintar, Joan Martínez-Campreciós, Sandra Aixut, Patricia Mossalilo, Elena Sulleiro, María Espiau, Santiago Mas-Coma, Ma. Dolores Bargues, Israel Molina

**Affiliations:** 1 Departamento de Parasitología, Facultad de Farmacia, Universidad de Valencia, Valencia, Spain; 2 Centro de Investigación Biomédica en Red de Enfermedades Infecciosas (CIBERINFEC), Instituto de Salud Carlos III, Madrid, Spain; 3 Tropical Medicine Unit Vall d’Hebron-Drassanes, Infectious Diseases Department, Vall d’Hebron University Hospital, PROSICS Barcelona, Barcelona, Spain; 4 Hospital Nossa Senhora da Paz, Cubal, Angola; 5 Department of Infectious Diseases, Vall Hebron Institut de Recerca (VHIR), Barcelona, Spain; 6 Microbiology Department, Vall d’Hebron University Hospital, PROSICS Barcelona, Barcelona, Spain; 7 Pediatric Infectious Diseases and Immunodeficiencies Unit, Department of Pediatrics, Vall d’Hebron University Hospital, PROSICS Barcelona, Barcelona, Spain; Federal University of Agriculture Abeokuta, NIGERIA

## Abstract

**Background:**

Schistosomiasis is one of the most important neglected tropical diseases, with a great impact on public health and more than 200,000 deaths annually. *Schistosoma haematobium* causes urinary tract (UT) morbidity. Since schistosomiasis morbidity control programs focus on children older than 5 years, pre-school age children (PSAC) morbidity is not well known.

**Methods:**

We conducted a cross-sectional study in Cubal (Angola) among 245 PSAC with the objective of evaluating the prevalence of *S*. *haematobium* infection, the intensity of infection, and associated morbidity. For this purpose, urine filtration test followed by microscopic visualization and ultrasound examinations were performed.

**Results:**

The estimated overall prevalence of urogenital schistosomiasis was 30.2% (CI 95%; 24.5–35.9), with 20.3% (CI 95%; 15.3–25.3) of the samples analysed showing a high intensity of infection. A total of 54.5% (CI 95%; 47.6–61.8) of infected children presented UT lesions, showing a significant association between schistosomiasis infection and UT morbidity (*p*-value < 0.001). Bladder wall thickening was the most common lesion, being present in 100% of abnormal ultrasounds. We found that anaemia and severe malnutrition were not significantly associated with the development of UT lesions.

**Conclusions:**

*S*. *haematobium* infection in PSAC causes great UT detectable morbidities. Therefore, there is an evident need of including them in mass drug administration (MDA) campaigns and consequently the development of an adapted praziquantel treatment dosage for children under 2 years of age.

## Author summary

Urogenital schistosomiasis caused by *Schistosoma haematobium* is a major public health problem in the impoverished population of tropical regions. The guidelines of the World Health Organization determine periodic mass drug administration with praziquantel as the main strategy to prevent morbidity associated with the chronic phase of the disease. This strategy has focused in school-age children, but recent recommendations suggest including other target populations, such as pre-school age children. In this study we evaluated the prevalence and morbidity associated with urogenital schistosomiasis in Cubal, an endemic area of Angola. We found a prevalence of urogenital schistosomiasis of 30.2% (CI 95%; 24.5–35.9) with a high infection intensity of 20.3% (CI 95%; 15.3–25.3) among pre-school age children. In addition, among the children infected, 54.5% (CI 95%; 47.6–61.8) presented urogenital abnormalities on ultrasound examination. There is an evident need of including them in mass drug administration campaigns and consequently the development of an adapted praziquantel treatment dosage for children under 2 years of age.

## Introduction

Schistosomiasis is a major public health problem in the impoverished population of tropical regions. It is the parasitic disease that causes the greatest morbidity after malaria, with 3.3 million Disability-Adjusted Life Years (DALY’s) and more than 200,000 deaths each year, 90% of which live in Sub-Saharan Africa [1,2].

Urogenital schistosomiasis is caused by the trematode *Schistosoma haematobium*, whose intermediate hosts are species of freshwater snails of the genus *Bulinus*. Humans become infected when they come into contact with freshwater sources contaminated with the free-swimming furcocercarial stage of the fluke. The adult worm lives in the venous plexus of the urinary bladder causing lesions to the kidney, bladder and ureter due to egg deposition [3] and the subsequent inflammatory reaction which, if persists, leads to fibrosis of the parenchymal tissue and can lead to bladder cancer in the long term.

Control of the disease is a challenge in the most affected regions due to many social activities carried out in contaminated water sources and the lack of universal access to safe water and sanitation. The World Health Organization (WHO) guidelines determine periodic mass drug administration (MDA) as the main strategy to prevent morbidity associated with the chronic phase of the disease, but it does not prevent reinfection. MDA is performed with a single 40 mg/kg dose of praziquantel, which guarantees adherence to treatment and facilitates administration logistics. In addition, the safety of the drug has been demonstrated in different risk groups such as school-age children (SAC), pre-school-age children (PSAC) or pregnant women [4–6].

Angola is an African country with an estimated population of 32.8 million, of which 49% are under 15 years old. According to the report made by the Ministério de Saúde de Angola in 2005, the national prevalence of urogenital schistosomiasis is 28% [7]. A recent study has shown a prevalence of almost 20% among SAC in the province of Benguela [8]. Previous investigations in the municipality of Cubal (Benguela) showed a prevalence of 61% among SAC and 85.3% of pathologic ultrasound findings, being the irregularities of the bladder wall, the distorted shape of bladder and the thickening of the wall the most common lesions [9,10]. The high prevalence of the disease at that age, along with the significant associated morbidity and the high percentage of people who are in contact with waterbodies in their daily activities, call for a better understanding of its epidemiology and its effects on the paediatric population. In addition, there is a specific concern about how the disease is affecting PSAC, since they are excluded from the MDA strategy, which is currently the only way to control the cumulative morbidity of this disease and is directed solely at SAC. Therefore, our objective is to analyse the prevalence and morbidity of urogenital schistosomiasis in children under 5 years of age.

## Methods

### Ethics statement

This study was approved by the ethics committee of the Ministério de Saúde de Angola (MINSA) (reference 41/2021). It was conducted in compliance with the principles of the Declaration of Helsinki, Good Clinical Practice guidelines, and local regulatory requirements. Parents or legal guardians of each child involved in the study were informed in Portuguese or Umbundu (local language) and gave written informed consent to participate. All data were anonymized and the study had no costs for the participants.

### Study design

A cross-sectional study was conducted between February and May 2022 in Cubal, located in west-central Angola (Angola). The municipality of Cubal is located in the province of Benguela, with an estimated population of 322,000 inhabitants, 47% of whom are under 15 and approximately 58,000 children are under 5 years of age [11]. It is a rural area that has several waterbodies used for human activities such as bathing or laundry. We identified three main freshwater hotspots with human activity. Two of them were part of the Cubal River, which crosses the municipality, and the other was a lake close to the town.

### Sample size calculation

The sample size was calculated based on an estimation of 58,000 PSAC, a local prevalence of schistosomiasis of 61% and urinary affectation of 85% according to a previous study by Bocanegra et al. [10]. Considering a 95% confidence interval [CI 95%], 7% precision and 10% loss, the sample size was estimated as 186 children under 5 years of age. Due to the fact that at the time of conducting the study we found an ultrasound dropout rate of 22.4%, the sample was increased to 245 children.

### Study population

The study was carried out in the facilities of Hospital Nossa Senhora da Paz in Cubal. As the target population was PSAC, traditional school recruitment was not possible. The community mobilization was done through the churches and markets, however, the recruitment of study participants was done at the hospital. The inclusion criteria were children under 5 years of age without symptoms suggestive of schistosomiasis. Participants were recruited from churches and the hospital (children accompanying relatives). The parents signed the informed consent. We excluded children whose parents (or legal guardians) objected to their participation. It should be noted that although the recruitment of PSAC was carried out in churches and in the hospital itself, both data collection and all clinical tests and analyses were carried out in the hospital facilities.

### Data collection

Epidemiological and clinical data were collected, including gender, age, neighbourhood, height, weight, mid-upper arm circumference (MUAC) and haemoglobin level. The degrees of malnutrition were determined according to the WHO standards: z-score values lower than -3 were classified as severe malnutrition, values between -2 and -2.99 as moderate, and values between -1.99 and -1 as mild malnutrition [12]. A urine sample was then collected and a urine dipstick test (Abbonn) was performed to describe the characteristics of the urine. In addition, an evaluation of the capillary haemoglobin (Hb) level was performed using the Haemocheck Kit TM (Remedy Healthcare). Anaemia was defined as haemoglobin values less than or equal to 11 g/dL, and values less than or equal to 7 g/dL were considered severe anaemia [13]. Finally, each participant was sent for a urological ultrasound examination.

### Urine examination

Researchers performed a preliminary evaluation of macroscopic haematuria by visual examination, followed by a urine dipstick test (Abbonn) to assess for the presence of leukocytes (trace for 15 cells/μl, one cross for 70 cells/μl, two crosses for 125 cells/μl and three crosses for 500 cells/μl or more), proteins (one cross for 0.3 g/L, two crosses for 1 g/L, three crosses for 3 g/L, and four crosses for 20 g/L or more) and erythrocytes (trace for less than 25 cells/μl, one cross for 25 cells/μl, two crosses for 80 cells/μl and three crosses for 80 cells/μl or more).

The presence of the eggs was evaluated with a laboratory magnifying glass after filtering the urine through 40 μm nylon filters (Thermo Fisher). The total number of eggs was also recorded to classify the intensity of infection according to the WHO standards, where less than 50 eggs/10 mL is considered to be low intensity and more than 50 eggs/10 mL is high intensity of infection [14].

### Urological ultrasound

All children were scheduled for urological ultrasound regardless of urine results. It was performed with a portable ultrasound machine (myLab 25; ESAOTE, Genova, Italy) at Hospital Nossa Senhora da Paz (Cubal) by a clinician trained both in the use of ultrasound machines and in the Niamey protocol and who was unaware of the rest of the analysis.

Urine pathological abnormalities were assessed following the Niamey protocol proposed by the WHO for the evaluation of *S*. *haematobium* infection [15]. The shape of the urinary bladder, the lesions detected in the bladder wall and the degree of dilatation of the ureters were recorded. The severity of the morbidity observed in the ultrasound was categorised using a global score based on each one of the pathological changes found according to the WHO score that classifies the affectation in scores from 0 to 32, with 0 being normal ultrasound and 1 in forward ultrasounds with abnormalities that increase the score according to the severity. This is the same methodology that was used in a previous study of urinary disorders caused by *S*. *haematobium* in SAC of Cubal [10].

### Statistical analysis

Quantitative variables were expressed as means and standard deviations (SD). Normally distributed quantitative variables were compared with the Student’s *t* test, and the Mann-Whitney test was used for non-normal variables. Categorical variables were compared using the Chi-square test (or Fisher’s exact test when the expected values were less than 5) and expressed as frequencies and percentages. Stepwise logistic regression analysis was used to calculate odds ratios (OR) and assess factors associated with infection and UT morbidity. Age, weight, height, z-score, and haemoglobin were considered as possible risk factors for *S*. *haematobium* infection. Macro-haematuria, micro-haematuria, leukocyturia, and proteinuria were the risk factors evaluated for possible association with UT morbidity. The level of significance for all tests was set at p< 0.05 with a 95% confidence interval (CI). All statistical analyses were performed using SPSS Statistics (Armonk, NY: IBM Corp).

Participants diagnosed with schistosomiasis received a single 40 mg/kg dose of praziquantel. Given the previously described high prevalence of schistosomiasis in the municipality, the single dose of the drug was administered after evidence of haematuria on the dipstick. Those who were diagnosed by the presence of *Schistosoma* eggs on the subsequent microscopic urinalysis received the drug after ultrasound testing. Those children with low values of haemoglobin or z-score were referred for further medical care.

All parents (or legal guardians) were informed about the results of the analysis and ultrasound scans as well as the epidemiology of the disease, along with a brief guide to prevent its spread.

## Results

### Demographic and clinical characteristics of the study population

A total of 245 children aged 5 or younger were included in the study: 106 (43.3%) girls and 139 (56.7%) boys. They lived in the Cubal district except for 18 children (7.3%) who lived in Caimbambo, a nearby rural village whose population often travels to Cubal in search of medical care. Their ages ranged from one month to 5 years, with a mean age of 3.3 years (SD 1.6). Haemoglobin values varied between 4 g/dL and 14 g/dL, with a mean value of 9.4 g/dL (SD 2.07). A total of 223 children (91.0%) (CI 95%; 87.4–94.6) presented anaemia and severe anaemia was detected in 57 children (23.3%) (CI 95%; 17.9–28.5). Regarding the nutritional status of the participants, 122 (49.8%) (CI 95%; 43.5–56.0) did not present malnutrition, 39 (15.9%) (CI 95%; 11.3–20.5) presented mild malnutrition and 23 (9.4%) (CI 95%; 5.74–13.0) moderate malnutrition, while 61 children (24.9%) (CI 95%; 19.5–30.3) suffered from severe malnutrition (z-score ≤ -3).

### Prevalence of *Schistosoma haematobium*

We detected the presence of *Schistosoma* eggs by microscopic examination in 74 (30.2%) (CI 95%; 24.5–35.9) children, of which 59 children presented low-intensity infection (79.7%) (CI 95%; 74.7–84.7) and 15 (20.3%) (CI 95%; CI 95%; 15.3–25.3) high-intensity infection. The mean age of the infected children was 3.1 years (SD 1.59). Age was associated with an increased likelihood of infection (OR 1.21 (1.02–1.43), *p*-value < 0.05). Furthermore, height (OR 1.03 (1.01–1.04), *p*-value < 0.05) and weight (OR 1.09 (1.03–1.16), *p*-value < 0.05) were also shown to be significantly associated with *S*. *haematobium* infection. No statistical differences were found with z-score and haemoglobin. There data are reported in Table 1.

**Table 1:** Demographic and clinical data for infected and non-infected children.

	Infected (N = 74)	Non-infected (N = 171)	OR (CI 95%)	*p*-value
Age (years)	3.1 (1.5)	2.8 (1.7)	1.21 (1.02–1.43)	<0.05
Weight (kg)	13.3 (4.5)	11.3 (4.6)	1.09 (1.03–1.16)	<0.05
Height (cm)	91.7 (15.5)	85.2 (15.2)	1.03 (1.01–1.04)	<0.05
Z-score	-1.2 (1.5)	-1.4 (1.6)	1.09 (0.91–1.30)	0.346
Haemoglobin	8.9 (1.9)	8.6 (2.1)	1.08 (0.94–1.23)	0.265

### Urine indirect test analysis

Leukocytes were detected in 70 (94.5%) (CI 95%; 91.7–97.4) samples, 20 with traces (27%), 25 (33.7%) with one cross, 14 (18.9%) with two crosses and 11 (14.86%) with three crosses. For erythrocytes, 59 (79.7%) (CI 95%; 74.7–84.7) samples were positive, 24 (32.4%) with traces, 10 (13.5%) with one and two crosses, and 15 (20.2%) with three crosses. Dipstick urinalysis showed that leukocytes and erythrocytes were found to be statistically associated with infection (OR 1.93 (1.39–2.68), *p*-value < 0.001). Proteinuria was positive on 19 (25.6%) (CI 95%; 20.1–31.1) samples, of which 6 (0.08%) presented traces, 8 (0.1%) one cross, 3 (0.04%) two crosses and 2 (0.02%) three crosses. Proteinuria was also statistically associated with the detection of *S*. *haematobium* eggs (OR 2.46 (1.42–4.24), *p*-value < 0.001). Macro-haematuria, which was present in 17 (22.9%) (CI 95%; 17.6–28.2) of the urines with *S*. *haematobium* eggs, was also shown to be significantly correlated with infection (OR 8.89 (2.79–28.34), *p*-value < 0.001).

### Ultrasound detectable morbidities

**Fig 1:**
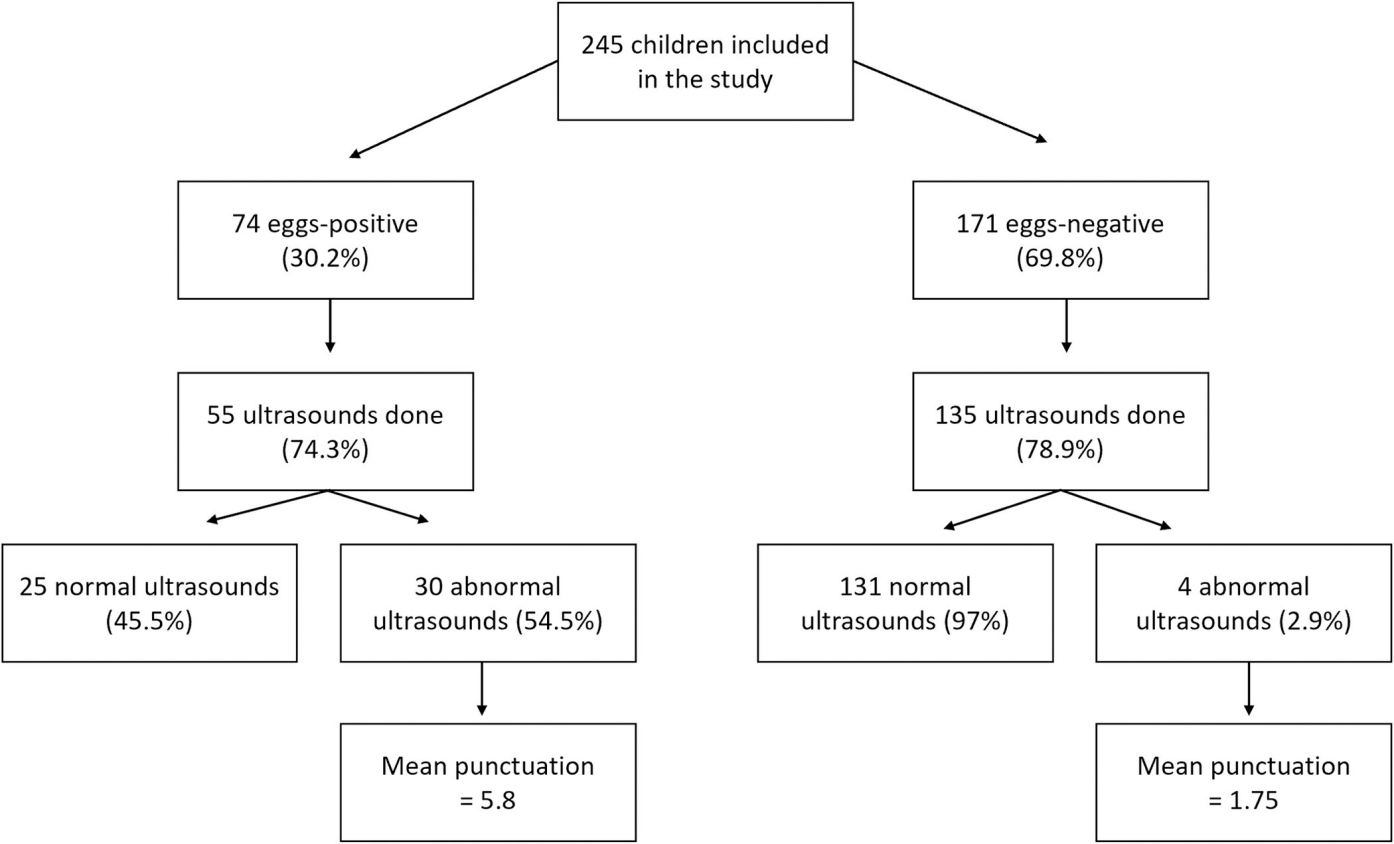
Distribution of children included in the current study.

As can be seen In Fig 1, urological ultrasound examination was performed on 190 children (77.5%), since the rest of the children missed the ultrasound appointment. Among the infected children, 30 (40.5%) (CI 95%; 29.3–51.7) presented at least one abnormality and had a mean score of 5.8 (SD 5.43). Regarding the ultrasound of the children without active infection, only 4 (2.9%) presented some abnormality, with a mean score of 1.75 (SD 0.43).

Table 2 reports the ultrasound results for the 30 children who had *S*. *haematobium* eggs. Bladder wall thickening was the most common finding (100% of abnormal ultrasounds) Seven (23.3%) (CI 95%; 8.20–38.4) had ureter abnormalities and 6 (20%) (CI 95%; 5.69–34.3) presented pelvis morbidity.

**Table 2:** Types of UT lesions in PSAC with active urogenital schistosomiasis.

	≤ 1 years old (N = 7)		2–3 years old (N = 7)		4–5 years old (N = 16)		Total (N = 30)	
Lesion	N (%)	OS	N (%)	OS	N (%)	OS	N (%)	OS
Bladder wall								
Wall irregularity	3 (42.9)	2	7 (100)	2	11 (68.8)	2	21 (70.0)	2
Wall thickening	7 (100)	2	7 (100)	2	16 (100)	2	30 (100)	2
Bladder masses	1 (14.3)	2	0 (0)	0	0 (0)	0	1 (3.3)	2
Pseudopolyp	0 (0)	0	0 (0)	0	0 (0)	0	0 (0)	0
Ureters								
Right ureter	1 (14.3)	4	1 (14.3)	3	1 (6.2)	3	3 (10.0)	3.3
Left ureter	2 (28.6)	3.5	0 (0)	0	3 (18.7)	3	5 (16.7)	3.2
Pelvis								
Right pelvis	1 (14.3)	8	0 (0)	0	1 (6.2)	8	2 (6.7)	8
Left pelvis	2 (28.6)	7	0 (0)	0	3 (18.7)	7.3	5 (16.7)	7.2
Mean score	7.8		4.1		5.8		5.8	

NOTE: Only children who underwent an abnormal ultrasound and had an ongoing *S*. *haematobium* infection are represented in the table. The number of children (N) with each affectation and their mean overall score (OS) stratified by age groups are shown. The number of children is reported next to the age title. The percentage (%) was calculated on the total number of children of the same age with ongoing *S*. *haematobium* and abnormal ultrasound

**Fig 2:**
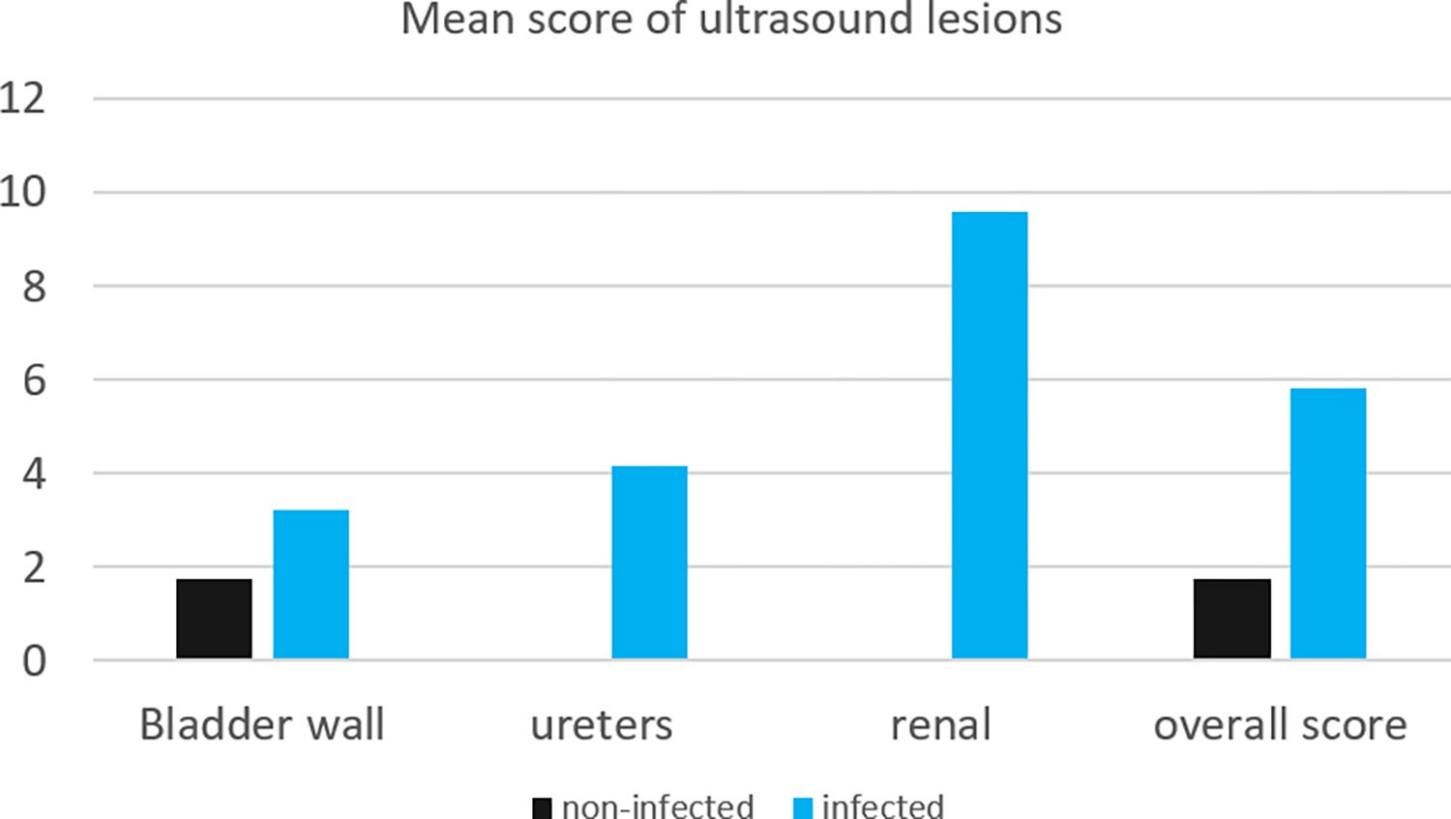
Plot of mean morbidity score for infected and uninfected children.

The mean morbidity score for the bladder wall, ureter, and kidneys, as well as the overall mean score, are shown in Fig 2. UT morbidity score was shown to be significantly correlated with schistosomiasis infection (OR 1.50 (6.51–34.56), *p*-value < 0.001).

The number of eggs found in the urine was higher in those children with abnormal ultrasound scans, showing a significant association between number of eggs in the urine and UT lesions (OR 3.69 (2.37–5.77), *p*-value < 0.001). As shown in Table 3, macro-haematuria (OR 4.14 (1.14–15.10), *p*-value < 0.05), micro-haematuria (OR 1.71 (1.29–2.26), *p*-value < 0.001) and proteinuria (OR 2.15 (1.14–4.08), *p*-value < 0.05) were correlated with UT morbidity. On the other hand, the statistical analysis showed that the development of UT lesions was not significantly correlated with anaemia (OR 1.08 (0.93–1.25), *p*-value = 0.264) but was significantly associated with severe malnutrition (OR 1.15 (0.91–1.44), *p*-value < 0.05).

**Table 3:** Prevalence of indirect morbidity assessment criteria in children with urinary tract morbidity and children with normal UT observed in ultrasound examination.

	Abnormal ultrasound (N = 34)	Normal ultrasound (N = 156)	OR (CI 95%)	*p*-value
Macro-haematuria	4 (11.7)	6 (3.8)	4.14 (1.14–15.10)	<0.05
Micro-haematuria	15 (44.1)	40 (25.6)	1.71 (1.29–2.26)	<0.001
Leukocyturia	17 (50.0)	25 (16.0)	1.28 (0.98–1.68)	0.076
Proteinuria	5 (14.7)	4 (2.5)	2.15 (1.14–4.08)	<0.05

## Discussion

Historically, schistosomiasis control and eradication programmes have focussed on SAC deworming in endemic countries. Targeting this age group leaves children under 5 years of age behind not only in MDA campaigns but also in research. Therefore, little is known about schistosome-related morbidity among the pre-school population. However, in recent years, some studies have been carried out to determine the prevalence of *S*. *haematobium* infection among PSAC, yielding data ranging from 0.83% to 74.9% [16–19]. As a consequence, different research groups demonstrated the efficacy and safety of praziquantel in PSAC and claimed for its inclusion in MDA campaigns [20]. It was in 2022 when the WHO modified its guide for MDAs against schistosomiasis and recommended applying it to children from 2 years of age [14]. Children under two years of age are also candidates for treatment on an individual basis, mainly due to the difficulty of adjusting the dose of praziquantel in this particular group. However, a suitable version of praziquantel for PSAC is currently under investigation [21].

Our study confirms that the high prevalence of urogenital schistosomiasis infection in PSAC also extends to the endemic areas of Angola with a prevalence of 30.2% (CI 95%; 24.5–35.9) in Cubal. It is within the expected values considering other prevalence studies in PSAC and previous research in the SAC group that was carried out in Cubal in 2014, where a prevalence of 61% was found [9]. The likelihood of infection increases with age, since older children have been in contact with infested water for a longer time. In fact, we found that age was correlated with the risk of infection. However, it is known that children in endemic areas are exposed to the parasite from the age of one year or less, since their mothers bathe them with river water [22].

An overwhelming 91% (CI 95%; 87.4–94.6) of children had anaemia, which is considerably higher than other studies on the burden of anaemia among African pre-school population [23,24], with 23.3% (CI 95%; 17.9–28.5) of these having severe anaemia. We did not find a significant association between anaemia and UT morbidity and there does not seem to be a significant correlation between anaemia and schistosomiasis infection, which could mean that these lesions are due to a chronic inflammation. This is understandable since anaemia is a multifactorial condition with a great impact in low- and middle-income countries where malnutrition, bacterial and parasitic infections (especially malaria), poor access to water and the use of unsanitary toilets have been correlated with low values of haemoglobin [25,26]. Severe anaemia can cause impaired physical and cognitive growth [27]. Regarding the level of malnutrition of the participants, 50.2% presented malnutrition, which is also above the nutritional values reported in studies carried out in other African countries where those levels ranged between 12 and 47.1% [28].

Detection of haematuria and proteinuria is commonly used as criteria to assess the severity of UT morbidity in *S*. *haematobium* infections. However, it is non-specific and may not correspond to reality (Table 3), so its exclusive use may be misleading. As an accurate assessment of morbidity, the use of ultrasonographic imaging is widely accepted [29]. The need to use ultrasound for the assessment of UT lesions gains importance since it remains asymptomatic until the disease progresses [30]. Our study revealed UT lesions in 54.5% (CI 95%; 47.6–61.8) of infected children, which is consistent with other studies of schistosomiasis morbidity [31,32] where PSAC shows a prevalence of UT morbidity associated with schistosomiasis of around 50%. According to our findings, we can state that children are affected with severe morbidity since 73.5% (CI 95%; 67.4–79.9) of abnormal ultrasounds had an overall score higher than 4. Surprisingly the highest score was obtained from a participant aged 10 months, with a score of 26. However, no precursor lesions of neoplasia such as pseudopolyp (0%) or masses in the bladder (2.9%) were found, which is consistent with the study by Barda et al. [32].

In our study, UT morbidity showed a significant association with schistosomiasis infection as only four abnormal ultrasounds were found in children with no *S*. *haematobium* eggs in the urine (2.5%) (Fig 2). However, it may be necessary to collect more than one sample in children with low egg excretion to be detected. Likewise, it has been pointed out that the absence of eggs in the urine may be due to the fact that they remain trapped in the bladder wall as a consequence of the inflammatory reaction, thus preventing their excretion [33].

MDA campaigns are the only tool available to prevent the cumulative morbidity caused by *S*. *haematobium* infection in endemic settings where access to health facilities may be a problem. Barda et al. [32] observed progression of UT pathology in 40% of children in just 6 months. This rapid evolution of morbidity raises the need to include PSAC in MDA campaigns. Although this is already contemplated in the new WHO guidelines for MDA programs in schistosomiasis [14], its recommendation is to cover children from two years of age, which excludes younger children who may suffer serious pathologies in the UT, as our study has shown. The neglect of these children and their non-treatment can lead to serious damage to the entire UT as well as irreversible damage to their integral development.

In summary, we have shown that infection by *S*. *haematobium* can occur as early as months of life and that there is a high UT morbidity in PSAC associated to urogenital schistosomiasis, especially thickening of the bladder wall. Consequently, the need to include them in MDA programs becomes evident.

The difficulty of the process for collecting urine samples in children under 1 year of age, which represents only 14.3% of the total number of PSAC included in the study, could lead to a possible bias. Furthermore, since urological ultrasound scans were performed in the hospital instead of at home with a portable ultrasound machine, only the parents (or legal guardians) most concerned about the health of their children attended the appointment, so it was not possible to assess the existence of pathological abnormalities in the urine in about 20% of children. Given that these are children whose parents are less concerned about their health, it can be assumed that the existence of UT lesions could be even much more common than in those children who attended the ultrasound scans. On the other hand, as the study was carried out in the municipality of Cubal, the study covers a small number of children from rural areas, so the findings cannot be easily generalized to the preschool population that lives far from the city and therefore with more difficulties in going to the hospital.

Despite these inherent limitations, the outcomes of our study highlight the need to include the paediatric population in MDA campaigns and intensify the diagnosis, management, and prevention of urogenital schistosomiasis in pre-school children.

## Supporting information

S1 Checklist STROBE Checklist. (DOC)
